# A Pilot Observational Study in Ohio, USA of the Healing of Our Veterans Equine Services Intensive Intervention for Veterans with Trauma Histories

**DOI:** 10.3390/healthcare13233111

**Published:** 2025-11-28

**Authors:** Amanda Held, Katy Hubbard, Elena Nazarenko, William Marchand

**Affiliations:** 1 H.O.O.V.E.S. (Healing of Our Veterans Equine Services), 4055 Wilkins Rd., Swanton, OH 43558, USA; amanda.h@hooves.us (A.H.);; 2 Whole Health Service, VA Salt Lake City Health Care System, 500 Foothill Drive, Salt Lake City, UT 84148, USA; 3 Department of Psychiatry, University of Utah, 501 Chipeta Way, Salt Lake City, UT 84108, USA; 4 Department of Animal, Dairy and Veterinary Sciences, Utah State University, 4815 Old Main Hill, Logan, UT 84322, USA

**Keywords:** equine-assisted services, psychotherapy incorporating horses, equine-assisted learning, military psychiatry, veterans, stress disorders, post-traumatic, trauma and stressor related disorders, depression, psychological flexibility

## Abstract

**Highlights:**

**What are the main findings?**

- The H.O.O.V.E.S. is an intensive equine-assisted learning program for veteran and active-duty military trauma survivors offered in a residential retreat format.
- Participation is associated with short-term increased psychological flexibility and positive affect as well as decreased anxiety, negative affect, depression and PTSD symptoms.

**What is the implication of the main finding?**

- The H.O.O.V.E.S. program has potential to benefit veteran and active-duty military trauma survivors.
- Further studies of this intervention are warranted.

**Abstract:**

**Background/Objectives**: Equine-assisted services are being increasingly utilized as complementary interventions for military veterans who have experienced trauma. However, rigorous research is lacking, and randomized controlled trials are needed. The H.O.O.V.E.S. Intensive intervention was developed for this population. This intensive program is an equine-assisted learning approach developed for veteran and active-duty military trauma survivors. The program integrates equine-assisted learning, peer mentorship and experiential learning in a residential retreat format. The primary aim of this pilot study was to determine if more rigorous studies of this intervention are warranted. Secondary aims were to assess preliminary outcomes and explore possible relationships between changes in outcome measures. **Methods**: This was a pilot prospective study. Inclusion criteria for the program included veteran or active-duty military status and a history of trauma exposure. Data were collected from April–October of 2024 in Ohio, USA. Six psychological instruments were administered to participants before, immediately after and 90 days and 120 days after the intervention. These were the PTSD Checklist for DSM-5 (PCL-5), the Acceptance and Action Questionnaire II (AAQII), the Positive and Negative Affect Scale (PANAS), the State-Trait Anxiety Inventory (STAI), the Beck Depression Inventory (BDI) and the Posttraumatic Growth Inventory (PTGI). Analyses were conducted to assess for significant changes across the study timeframe and for relationships among the changes in psychological instrument scores. **Results**: Study participants were 32 veterans with trauma histories ranging in age from 30 to 67 years old. There were statistically significant pre- to post-intervention improvements for all instruments except the PTGI, suggesting short-term increased psychological flexibility (AAQII) and positive affect (PANAS-positive) as well as decreased anxiety (STAI), negative affect (PANAS-negative), depression (BDI) and PTSD symptoms (PCL-5). Changes in BDI and PCL-5 scores persisted at 120 days post-intervention whereas changes in the AAQII, PANAS and STAI did not persist. Increased psychological flexibility was correlated with reductions in negative affect, PTSD symptoms and anxiety, as well as with increases in positive affect. **Conclusions**: Preliminary results reported herein suggest participation is associated with psychological benefits. Further, decreased experiential avoidance/increased psychological flexibility should be explored as an underlying mechanism potentially contributing to the benefits of participation in EAS. A randomized controlled trial of the H.O.O.V.E.S. Intensive program is warranted.

## 1. Introduction

Military veterans frequently have trauma exposure histories. Rates of posttraumatic stress disorder (PTSD) approach 30% [1,2] and up to 15% of female veterans have experienced military sexual trauma [3]. Further, elevated depression and anxiety symptoms have been found in 11.0 and 9.9% of veterans, respectively [4]. PTSD is associated with impairment in social, occupational and physical functioning including quality of life and physical health problems [5] along with suicidal ideation, attempts and death from suicide [5]. Effective interventions are available [6]; however, these conventional approaches have high attrition and relatively low response rates [7,8,9]. Further, these approaches may not fully address the complex aftereffects of military sexual trauma [10].

In response to the above, complementary interventions for this population have been explored with the aim of enhancing treatment engagement and/or outcomes [11,12] as well as improving well-being and resilience in veterans. Equine-assisted services (EAS) are complementary animal-assisted interventions aimed at providing benefits for human participants [13]. EAS are being increasingly used for both civilian [14] and military [15,16,17,18,19] trauma survivors. Specific approaches include psychotherapy incorporating horses (PIH), equine-assisted learning (EAL) and therapeutic riding [13].

The field of EAS is in the early stages of scientific development and, as we have previously reviewed, the psychological mechanisms underlying potential benefits are not fully elucidated [20]. Further, it is likely that various mechanisms of action function simultaneously and synergistically [20]. Hypothesized potential mechanisms include the development of horse–human relationships including attachment and bonding, an enhanced sense of control, autonomy and assertiveness for participants, decreased arousal, emotional mirroring, self-distancing through metaphor, biophilia, and enhanced psychological flexibility and mindfulness [20]. Additionally, potential outcomes associated with participation are still under investigation [20]. However, the literature indicates improvements in anxiety and/or depression/affect in veteran [15,17,21,22,23,24,25,26,27,28,29,30,31] populations. Further, decreased PTSD symptoms have also been reported in both community [14,32] and veteran [15,17,18,19,21,22,26,28,30,31,33,34,35,36,37] populations. While promising, these findings are difficult to interpret due to both a lack of rigorous studies and a lack of standardization of interventions and terminology [20]. Thus, additional research is needed, in particular randomized controlled trials.

We were particularly interested in psychological flexibility (PF) for which we have previously found evidence of increases associated with EAS participation in four investigations [28,29,38,39]. PF is defined as the continued pursuit of valued goals despite experiencing distress [40] and is positively related to resilience [41,42,43], overall psychological health [42] and self-efficacy [44]. Experiential avoidance (EA) is the opposite of PF and is the tendency to avoid, suppress or control unwanted internal experiences [45]. There is evidence that EA plays a role in substance use disorders, posttraumatic psychological distress [46] and symptom severity in some disorders such as generalized anxiety disorder [41]. Thus, we have hypothesized that increased PF may be an important mechanism underlying benefits associated with EAS and therefore, understanding the relationship between changes in PF and other outcome variables will make an important contribution to the literature.

The Healing Of Our Veterans Equine Services (H.O.O.V.E.S.) Intensive program is an EAL approach developed for veteran and active-duty military trauma survivors. The program integrates equine-assisted learning, peer mentorship and experiential learning in a residential retreat format. The rationale behind the intervention is grounded in the understanding that horses, as highly sensitive prey animals, respond to human emotional states and can act as mirrors for internal experiences. This unique feedback facilitates self-awareness, emotional regulation, and authentic connection. The program was designed to complement conventional treatment approaches while addressing high attrition rates and barriers to engagement seen in veteran populations. The H.O.O.V.E.S. Intensive program, with its integrated and retreat-based format, differs conceptually from many EAS programs for veterans. Currently there is a lack of standardization of interventions in the field, and a wide variety of programming is offered [20]. A recent review [20] found that among 23 papers describing programming for veterans, the majority, 16 interventions, were provided in a format of weekly sessions of varying numbers and only four used a retreat or continuous multi-day format.

The primary aim of this observational pilot study was to determine if a randomized controlled trial (RCT) of the H.O.O.V.E.S. Intensive intervention for veterans is warranted. Secondary aims were to assess the benefits of the unique retreat-based format for this population, evaluate potential psychological instruments for use in a future RCT, and to assess preliminary outcomes and explore possible relationships between changes in PF and other outcome measures.

Key findings of this study indicate that H.O.O.V.E.S. participation is associated with short-term increased psychological flexibility and positive affect as well as decreased anxiety, negative affect, depression and PTSD symptoms. Changes in depression and PTSD symptoms persisted at 120 days post-intervention. Increased psychological flexibility was correlated with reductions in negative affect, PTSD symptoms and anxiety, as well as with increases in positive affect.

## 2. Materials and Methods

### 2.1. Intervention

The H.O.O.V.E.S. Intensive is a five-day residential program that combines equine-assisted learning, peer support and evidence-based self-discovery practices. This intensive program uses a standardized protocol guided by a 100-page manual, ensuring consistency across programs. Facilitator training is standardized and follows the three-level Equine Wisdom Integration Method. Veterans live onsite at the H.O.O.V.E.S. Sanctuary, where they participate in daily equine-guided activities, group processing sessions and individual reflection exercises. Core components include groundwork with horses, the Human Blueprint exercise and integration activities designed to translate equine feedback into actionable personal insights. Participants also engage in physical activities such as the Miles 2 Freedom cycling initiative, mindfulness practices and community meals, creating a holistic and immersive healing environment. Each program serves 8–10 veterans, with alumni mentors supporting participants throughout. The Intensive program is offered seven times per year.

### 2.2. Study Design

This study was an uncontrolled and nonrandomized pilot observational investigation of the H.O.O.V.E.S. Intensive program, which was being provided for clinical, not research, purposes. The program was provided in the state of Ohio, USA. Data was collected for the intensive programs offered 23–28 April, 21–26 May, 16–21 July, 27 August–1 September and 1–6 October 2024.

Subjects for this study were a convenience sample of veterans who enrolled in one of five H.O.O.V.E.S. Intensive programs offered in 2024. Recruitment occurs through referrals from the Veterans Health Administration (VHA), veteran service organizations, word-of-mouth and direct outreach via the H.O.O.V.E.S. website and social media channels. Interested veterans complete an application that includes demographic and background information. Applicants are contacted by staff for an intake call to assess readiness and ensure program fitness. Inclusion criteria for the program included veteran or active-duty military status and a history of trauma exposure. Though exclusion criteria were limited, individuals with active psychosis, active substance use or unmanaged violent behavior would be deferred until stabilized. For the study, inclusion criteria were enrollment in the intensive program and voluntary agreement to participate in the research project. Declining research participation did not impact program acceptance or participation. Individuals who enrolled in the program were given the option to participate in the research project. They were informed that declining to participate would have no negative repercussions and that they would receive the same programming as those who participated in the research. The inclusion criteria for the study were enrollment in the intensive program and desire to participate in the research. There were no additional exclusion criteria for the research project. Those who elected to participate were sent an electronic message with a cover letter describing the research and giving them an opportunity to participate. Electing to participate indicated implied informed consent.

### 2.3. Psychological Instruments

Six psychological instruments were utilized to assess for changes associated with program participation. Instruments were chosen to align with the literature review and hypotheses stated in the introduction. These were the PTSD Checklist for DSM-5, the Acceptance and Action Questionnaire II, the Positive and Negative Affect Scale, the State-Trait Anxiety Inventory and the Beck Depression Inventory. Additionally, the Posttraumatic Growth Inventory was administered for exploratory analyses as to our knowledge, this instrument has not been previously used in EAS research.

The PTSD Checklist for DSM-5 (PCL-5) is a 20-item English language instrument used to evaluate PTSD symptom severity [47]. The questions are in a 5-point Likert scale format that range from 0 to 4. Total scores range from 0 to 80 with higher scores corresponding to greater symptoms of PTSD. Psychometric properties include good internal consistency across samples and diagnostic validity [48]. The Acceptance and Action Questionnaire II (AAQII) measures psychological flexibility (PF) and experiential avoidance [49]. This English language instrument uses a 10-item, 7-point Likert scale. Scores range from 10 to 70 and lower scores indicate greater PF and decreased experiential avoidance. Psychometric properties include good internal consistency but only fair test-retest and discriminate validity [49]. The Positive and Negative Affect Scale (PANAS) assesses affect. There are 20 positive and negative affect statements on a 5-point Likert English language scale [50]. Each component of the PANAS score ranges from 10 to 50. Greater positive emotion is indicated by higher scores on positive affect (PANAS-positive) and higher scores on negative affect (PANAS-negative) signify greater negative emotion. Psychometric properties include good internal consistency and test-retest reliability as well as a robust two-factor structure [50]. The State-Trait Anxiety Inventory (STAI) is a 6-item English language instrument that measures state anxiety [51] with higher scores indicating greater state anxiety. Psychometric properties include excellent internal consistency and sensitivity to change but lower test-retest reliability [52]. The Beck Depression Inventory (BDI) is a 21-item English language instrument that assesses depressive symptoms on a 4-point Likert scale [53]. Scores range from 0 to 63, with higher scores indicating greater levels of depression. Psychometric properties include excellent internal consistency and convergent validity as well as sensitivity to change [53]. Finally, the Posttraumatic Growth Inventory (PTGI) assesses post-trauma growth and self-improvement [54]. It is a 21-item English language scale that assesses five factors: Personal Strength; New Possibilities; Improved Relationships; Spiritual Growth; and Appreciation for Life. Participants indicate their scores on a 6-point scale where, 0 = “I did not experience this as a result of my crisis”; 1 = “I experienced this change to a very small degree as a result of my crisis”; 2 = “I experienced this change to a small degree as a result of my crisis”; 3 = “I experienced this change to a moderate degree as a result of my crisis”; 4 = “I experienced this change to a great degree as a result of my crisis”; and 5 = “I experienced this change to a very great degree as a result of my crisis.” Higher total scores indicate positive transformation. Psychometric properties include good to excellent internal consistency and adequate temporal stability [54]. The AAQII, PANAS and STAI were administered at 10 timepoints, at the beginning and end of the intervention as well as before and after each daily session. The PCL-5, PTGI and BDI were administered at four time points, pre-, immediate post-, 90 days post- and 120 days post-intervention.

### 2.4. Data Handling and Analyses

All data was collected by electronic surveys utilizing the VHA Research Electronic Data Capture (REDCap) platform [55], a secure web-based platform designed to support data capture for research studies.

To evaluate for significant changes in psychological instrument scores associated with participation, one-tailed paired *t*-tests were utilized to compare pre- to immediate post-intervention outcomes. Additionally, a preliminary analysis was conducted to assess whether changes persisted at 90 and 120 days post-intervention for those instruments with statistically significant pre- to post-intervention changes (PCL-5 and BDI). Changes in scores across the four time points were analyzed using the Friedman test for related samples. Only participants with complete data at all four time points were included in the analysis (complete-case analysis). Where the Friedman test was significant, post hoc pairwise comparisons were conducted using the Wilcoxon signed-rank test. Bonferroni correction was applied to adjust for multiple comparisons. Effect sizes for significant Wilcoxon tests were calculated, where Z is the standardized test statistic and N is the number of observations.

To evaluate whether changes across psychological measures were related, we first calculated change scores for each participant by subtracting pre-treatment scores from post-treatment scores (Δ = post − pre). Negative values indicated symptom reduction (e.g., anxiety, depression, PTSD, negative affect, experiential avoidance), while positive values indicated improvement in positive affect. We then conducted bivariate correlations among all change scores using Pearson’s r as the primary statistic, as this is the conventional measure of linear association in psychological research. Because of the modest sample size and potential departures from normality, we also computed Spearman rank-order correlations (ρ) as a non-parametric robustness check; results were highly consistent across the two approaches. To reduce the risk of Type I error, all correlation *p*-values were corrected for multiple comparisons using the false discovery rate (FDR) procedure. To examine whether changes in one measure could uniquely predict changes in another when accounting for overlap across instruments, we conducted a series of multiple linear regression models. For each outcome (e.g., ΔSTAI, ΔPCL) all other change scores were simultaneously entered as predictors. Standardized beta coefficients (β) are reported to facilitate comparison across measures. Regression *p*-values were also corrected within each outcome using the FDR procedure. Both the full regression models and the strongest unique predictors for each outcome are reported.

### 2.5. Approvals

This study was approved by the affiliate university institutional review board (IRB_00166616) and the VA facility Research and Development Committee as exemption Category 2, including approval of waiver/alteration of informed consent. The equines were providing regular clinical services. No animal-related research data was collected, thus Institutional Animal Care and Use Committee approval was not required. This study was also registered with ClinicalTrials.gov, registration number NCT06300255.

## 3. Results

There were 38 individuals enrolled in the intensive program. Of these, 84.2% elected to participate in the research project. The final number of study participants was 32 veterans ranging in age from 30 to 67 years old. They were residents of 16 different states, including five from Ohio. See Table 1 for demographic and diagnostic data (this information was missing for one participant). Among the participants 65% were currently receiving psychotherapy and 45% were receiving psychopharmacology. Mean immediate pre-intervention scores of the PCL-5 and BDI (Table 2) were 55.12 and 29.36, respectively, indicating that most participants had probable PTSD and were experiencing moderate depressive symptoms.

There were statistically significant (see Table 2 for immediate pre- to immediate post-intervention changes) pre- to post-intervention improvements in scores for all instruments except the PTGI. Effect sizes were large to very large except for the PANAS positive, which was moderate. Results for the preliminary analyses over four time periods (pre-, immediate post-, 90 days post- and 120 days post-intervention) for the PCL-5 and BDI indicated significant changes from pre- to 120-days post- for both. There were no significant changes for any other instruments across time (see Table 3 for immediate pre- to 120 day post-intervention changes).

Change scores (post-treatment minus pre-treatment) were calculated for each measure, with negative scores reflecting either symptom reduction (PANAS-N, STAI, PCL, BDI) or decreased experiential avoidance (AAQII) and positive scores reflecting gains in positive affect (PANAS-P) (see Table 4 for Pearson correlations of these change scores). Spearman correlations were also computed and produced a nearly identical pattern of results, confirming robustness. Several clear relationships emerged. Greater reductions in anxiety (STAI) were strongly associated with greater reductions in negative affect (PANAS-N) and PTSD symptoms (PCL), as well as greater increases in positive affect (PANAS-P). Reductions in experiential avoidance (AAQII) were also correlated with reductions in negative affect, PTSD symptoms and anxiety, as well as with increases in positive affect. Depression (BDI) and PTSD symptoms (PCL) showed a particularly strong correlation, indicating that improvements in depression tended to co-occur with improvements in PTSD. When examining unique predictors using multiple regression changes in depression (BDI) significantly predicted changes in PTSD symptoms (PCL), and vice versa, even after adjusting for all other measures. Although changes in anxiety, affect and experiential avoidance were highly interrelated, none emerged as unique predictors once the overlap among measures was accounted for (see Table 5 for the strongest predictor of each change score outcome).

## 4. Discussion

The primary aim of this observational pilot study was to determine if a randomized controlled trial of the H.O.O.V.E.S. Intensive intervention for veterans is warranted. Secondary aims were to evaluate potential psychological instruments for use in such a study and to assess preliminary outcomes along with disambiguating possible relationships between changes in PF and other outcome measures.

Results revealed significant pre- to post-intervention changes in psychological test scores for all instruments except the PTGI (see Table 2 for pre- to post-intervention changes). These changes suggest short-term increased PF (AAQII) and positive affect (PANAS-positive) as well as decreased anxiety (STAI), negative affect (PANAS-negative), depression (BDI) and PTSD symptoms (PCL-5). Effect sizes were large to very large except for the PANAS positive, which was moderate. Preliminary analyses (Table 3) suggested that BDI and PCL-5 scores remained significantly lower than pre-intervention at 120 days post-intervention. Further, preliminary analyses were conducted (Table 4 and Table 5) to begin to disambiguate relationships between pre- to post-intervention changes in psychological instrument scores. Reductions in EA were correlated with reductions in negative affect, PTSD symptoms and anxiety, as well as with increases in positive affect. Lastly, multiple regression analyses indicated changes in depression (BDI) significantly predicted changes in PTSD symptoms (PCL) and vice versa.

Findings regarding depressive and anxiety symptoms are consistent with the literature reports of improvements in anxiety and/or depression/affect in community [56,57] and veteran [15,17,21,22,23,24,25,26,27,28,29,30,31] populations associated with various EAS interventions. Further, decreased PTSD symptoms have also been reported in both community [14,32] and veteran [15,17,18,19,21,22,26,28,31,33,34,35,36,37] populations. Findings of increased PF/decreased experiential avoidance are also consistent with the literature as these findings have been previously reported in both veteran [28,29] and non-veteran [38,39] populations. The finding that reductions in EA were correlated with other outcomes is consistent with the literature indicating that EA is a core mechanism in the development and maintenance of anxiety and disrupts pleasant activity [41] as well as contributes to the severity of PTSD symptoms [46].

The degree of change in depression and anxiety symptoms was particularly notable. The mean pre-intervention BDI score was 29 (Table 2) suggesting participants were experiencing moderate to severe depressive symptoms [53]. The post-intervention mean was 8, which is consistent with minimal depression [53] and the mean remained significantly lower than pre- at 120 days post-intervention (Table 3). Similarly, regarding the PCL-5, the mean pre-intervention score was 55 (Table 2) indicating that most participants had probable PTSD [48]. In contrast, the mean post-intervention score dropped to 25, which is below the cutoff for probable PTSD [48]. Further, there was a ≥20-point decrease in mean scores, which is often considered very strong evidence of meaningful clinical change [47]. Also, PCL-5 scores also remained significantly lower than pre-intervention at 120 days post-intervention. Lastly, the finding that changes in depression significantly predicted changes in PTSD symptoms and vice versa is not surprising given that PTSD and depression are highly comorbid and greater PTSD symptom severity is strongly associated with greater depressive symptoms when the conditions co-occur [58].

As stated in the introduction, the field of EAS is in the early stages of scientific development and the potential benefits and the underlying psychological mechanisms are not fully understood [20].

To begin to address gaps in the literature, we were particularly interested in PF, as measured by the AAQII. As stated in the introduction, PF is defined as the continued pursuit of valued goals despite experiencing distress [40] and is positively related to resilience [41,42,43], psychological health [42] and self-efficacy [44]. PF is the opposite of EA which is the tendency to avoid, suppress or control unwanted internal experiences [45]. Decreased AAQII scores, as were found in this and our previous studies [28,29,38,39], indicate increased PF and decreased EA [49]. Given the consistency of these findings and evidence that EA plays a role in substance use, anxiety [41] and posttraumatic stress disorders [46], we have hypothesized that decreased EA/increased PF may be an important mechanism underlying some benefits associated with EAS. Thus, understanding the relationship between changes in PF and other outcome variables may make an important contribution to the literature. In this study, reductions in EA were correlated with reductions in negative affect, PTSD symptoms and anxiety, as well as with increases in positive affect. If replicated by more rigorous studies, these results suggest that transdiagnostic changes in PF/EA might underlie some psychological benefits associated with EAS participation.

If reduced EA is a mediator of some of the benefits of EAL, future studies will need to explore the mechanisms underlying changes in PF/EA associated with EAS in this and other interventions. In other words, how does EAL participation result in more willingness to experience difficult thoughts, emotions, memories or sensations without trying to suppress them? There is a close relationship between PF and mindfulness [59] and experiential avoidance is an important mediator for alleviating emotional distress through mindfulness practice [60]. Thus, mindfulness might play a role in the association between EAL and reduced EA. The H.O.O.V.E.S. Intensive intervention includes meditation practice, and some interventions [29] focus specifically on teaching mindfulness skills. However, beyond mindfulness training incorporated into EAS, it has been hypothesized that animal-assisted interventions can be an effective form of mindfulness training [61] and working with large animals, such as horses provides a compelling reason to focus on the present [62]. Lastly, nature exposure, or biophilia, may play a role in impacting PF/EA. Studies [63,64] of nature exposure for veterans have also reported enhanced PF and nature exposure is commonly associated with benefits similar to those associated with increased PF, including enhanced resilience [65]. Future investigations of EAS mechanisms of action should measure changes in mindfulness and PF/EA during different activities to disambiguate the potential role of these psychological constructs in the benefits of EAL.

In summary, while this was a pilot study with many limitations, these results are very promising and indicate a randomized controlled trial of this intervention is warranted and that the psychological instruments used in this study are appropriate for such a study. Also, further explorations of EA as a mediator of some benefits of EAL are warranted.

Limitations that must be considered when evaluating these results include the lack of randomized and control condition and utilization of a convenience sample. Thus, selection bias is a concern and cause and effect relationships were not demonstrated. Also, treatment fidelity was not monitored. Other limitations were reliance on self-report measures and that attrition across timepoints and temporal trends were not analyzed beyond pairwise comparisons. Additionally, the number of instruments utilized may have resulted in participant fatigue and thus unstable and/or inaccurate responses. Further, the sample size was small particularly for 90 and 120 day post-intervention results. Future studies should include a control or waitlist group, recruit larger and more homogeneous samples and monitor concurrent treatments to reduce confounding factors. Future research should also include performing and reporting a trend analysis to capture within-subject changes more precisely. Nonetheless, this study achieved the aim of determining that further studies of this intervention are warranted.

## 5. Conclusions

The H.O.O.V.E.S. Intensive program shows promise as a complementary intervention for veterans who have experienced trauma. Preliminary results reported herein suggest participation is associated with increased positive affect and psychological flexibility as well as decreased anxiety, negative affect, depression and PTSD symptoms. Improvements in depressive and PTSD symptoms may persist for 120 days post-intervention. Further, decreased experiential avoidance/increased psychological flexibility should be explored as an underlying mechanism associated with the benefits of participation in EAS. A randomized controlled trial of the H.O.O.V.E.S. Intensive program is warranted.

## Figures and Tables

**Table 1:** Demographic, diagnostic and treatment characteristics of participants (n = 31).

		Range, Mean (SD)	Number (%)
Age		30–67, 48.3 (9.9)	
Gender	Male		8 (26%)
	Female		23 (74%)
Race	White		18 (58%)
	Black		9 (29%)
	American Indian/Alaksa Native		2 (7%)
	Asian		1 (3%)
	Native Hawaiian or Other Pacific Islander		1 (3%)
Ethnicity	Not Hispanic or Latino		25 (81%)
	Hispanic or Latino		6 (19%)
Religion	Christianity		22 (71%)
	Other/none		9 (29%)
Relationship Status	Married		13 (42%)
	Divorced/separated		10 (32%)
	Single		5 (16%)
	Living with a partner, non-married		2 (7%)
	Widowed		1 (3%)
Military-related disability	Yes		29 (93%)
	No		2 (7%)
Percent disabled rating		0–100, 77 (34.9)	
Mental health diagnoses	PTSD		19 (61%)
	Alcohol use disorder		5 (16%)
	Other substance use disorder		4 (13%)
	Any mood disorder		12 (39%)
	Other mental health condition		7 (23%)
Combat-related trauma			9 (29%)
Military trauma not combat-related			13 (42%)
MST			21 (68%)
History of childhood abuse/neglect			18 (58%)
History of adult non-military trauma			20 (65%)
Current mental health treatment	Psychotherapy		20 (65%)
	Psychopharmacology		14 (45%)
	None		8 (26%)
History of inpatient mental health treatment			9 (29%)

MST = military sexual trauma; PTSD = posttraumatic stress disorder.

**Table 2:** Immediate pre- to immediate post-intervention outcomes.

Instrument	Pre-Intervention	Post-Intervention	Statistics
AAQII (n = 27)	M = 36.6 (7.3)	M = 21.4	*t* (26) = 9.33
	SD = 7.3	SD = 8.9	*p* < 0.001 *
			*d* = 1.8
PANAS-N (n = 27)	M = 32.78	M = 18.26	*t* (26) = 6.30
	SD = 9.31	SD = 9.81	*p* < 0.001 *
			*d* = 1.2
PANAS-P (n = 27)	M = 29.41	M = 34.30	*t* (26) = 2.38
	SD = 7.52	SD = 10.22	*p* = 0.01 *
			*d* = 0.46
STAI (n = 27)	M = 57.96	M = 36.11	*t* (26) = 6.57
	SD = 15.65	SD = 13.20	*p* < 0.001 *
			*d* = 1.26
PCL-5 (n = 25)	M = 55.12	M = 25.00	*t* (24) = 7.81
	SD = 14.47	SD = 16.80	*p* < 0.001 *
			*d* = 1.56
PTGI (n = 25)	M = 45.92	M = 57.56	*t* (24) = −1.69
	SD = 23.12	SD = 34.67	*p* = 0.948
			-
BDI (n = 22)	M = 29.36	M = 8.23	*t* (21) = 6.83
	SD = 10.67	SD = 9.85	*p* < 0.001 *
			*d* = 1.46

* = statistically significant result; AAQII = Acceptance and Action Questionnaire II; PANAS-P = Positive and Negative Affect Scale—positive affect; PANAS-N = Positive and Negative Affect Scale—negative affect; STAI = State-Trait Anxiety Inventory; PCL-5 = PTSD Checklist for DSM-5; PTGI = Posttraumatic Growth Inventory; BDI = Beck Depression Inventory.

**Table 3:** Immediate pre- to 120 day post-intervention preliminary outcomes.

Instrument	Time Point	Mean (SD)	Friedman Test	Wilcoxon Comparison	Wilcoxon *p*	Bonferroni *p*	Effect Size r
PCL-5 (n = 13)	Pre	51.46 (17.56)	χ^2^ (3) = 14.07 *p* = 0.0028	Pre vs. Immediate Post	0.0029	0.0172 *	0.827
	Immediate post	27.92 (18.97)	-	Pre vs. Post 90 Days	0.0398	0.2388	-
	90 days post	32.69 (26.02)	-	Pre vs. Post 120 Days	0.0012	0.0073 *	0.897
	120 days post	21.46 (10.42)	-	Immediate Post vs. Post 90 Days	0.6848	1.0	-
	-	-	-	Immediate Post vs. Post 120 Days	0.3054	1.0	-
	-	-	-	Post 90 Days vs. Post 120 Days	0.1677	1.0	-
BDI (n = 9)	Pre	25.89 (11.31)	χ^2^ (3) = 12.17 *p* = 0.0068	Pre vs. Immediate Post	0.0078	0.0469 *	0.887
	Immediate post	7.44 (8.17)	-	Pre vs. Post 90 Days	0.0742	0.4453	-
	90 days post	15.78 (15.06)	-	Pre vs. Post 120 Days	0.0039	0.0234 *	0.962
	120 days post	12.0 (9.47)	-	Immediate Post vs. Post 90 Days	0.2076	1.0	-
	-	-	-	Immediate Post vs. Post 120 Days	0.2603	1.0	-
	-	-	-	Post 90 Days vs. Post 120 Days	0.4258	1.0	-

* = statistically significant result after correction for multiple comparisons; PCL-5 = PTSD Checklist for DSM-5; BDI = Beck Depression Inventory.

**Table 4:** Correlations among pre- to post-treatment change scores.

Change-Score Pair	n	Pearson *r*	Adj. *p* (FDR)
ΔAAQII ↔ ΔBDI	22	0.24	0.301
ΔAAQII ↔ ΔPANAS-N	27	0.66	0.001 *
ΔAAQII ↔ ΔPANAS-P	27	−0.51	0.012 *
ΔAAQII ↔ ΔPCL	25	0.47	0.028 *
ΔAAQII ↔ ΔSTAI	27	0.57	0.004 *
ΔBDI ↔ ΔPANAS-N	22	0.21	0.393
ΔBDI ↔ ΔPANAS-P	22	−0.34	0.190
ΔBDI ↔ ΔPCL	20	0.73	0.001 *
ΔBDI ↔ ΔSTAI	22	0.42	0.073
ΔPANAS-N ↔ ΔPANAS-P	27	−0.46	0.028 *
ΔPANAS-N ↔ ΔPCL	25	0.59	0.004 *
ΔPANAS-N ↔ ΔSTAI	27	0.78	<0.001 *
ΔPANAS-P ↔ ΔPCL	25	−0.30	0.158
ΔPANAS-P ↔ ΔSTAI	27	−0.66	0.001 *
ΔPCL ↔ ΔSTAI	25	0.70	0.001 *

Negative correlations indicate that greater improvements (reductions) in one measure were associated with greater improvements in another. *p*-values are adjusted for multiple comparisons using the false discovery rate (FDR). Δ = change; * = statistically significant result; AAQII = Acceptance and Action Questionnaire II; PANAS-P = Positive and Negative Affect Scale—positive affect; PANAS-N = Positive and Negative Affect Scale—negative affect; STAI = State-Trait Anxiety Inventory; PCL-5 = PTSD Checklist for DSM-5; BDI = Beck Depression Inventory.

**Table 5:** Condensed multiple regression results: strongest predictor of each change score outcome.

Outcome (Δ)	Strongest Predictor (Δ)	Standardized β	Adj. *p* (FDR)	n	R^2^	Adj. R^2^
ΔAAQII	ΔPCL	0.36	0.542	20	0.64	0.52
ΔBDI	ΔPCL	0.91	0.011 *	20	0.63	0.50
ΔPANAS-N	ΔPCL	0.53	0.174	20	0.65	0.53
ΔPANAS-P	ΔPCL	−0.44	0.267	20	0.58	0.43
ΔPCL	ΔBDI	0.55	0.011 *	20	0.77	0.69
ΔSTAI	ΔPANAS-N	0.44	0.265	20	0.84	0.79

Δ = change; * = statistically significant result after correction for multiple comparisons; AAQII = Acceptance and Action Questionnaire II; PANAS-P = Positive and Negative Affect Scale—positive affect; PANAS-N = Positive and Negative Affect Scale—negative affect; STAI = State-Trait Anxiety Inventory; PCL-5 = PTSD Checklist for DSM-5; BDI = Beck Depression Inventory.

## Data Availability

The data underlying this study are not publicly available due to U.S. Department of Veterans Affairs (VA) regulations and federal privacy laws protecting sensitive veteran information. Access to data may be granted to qualified researchers with appropriate VA approvals, including Institutional Review Board (IRB) review and a signed data use agreement. Requests for data access may be directed to the VA Office of Research and Development or the corresponding author.

## Abbreviations

The following abbreviations are used in this manuscript:

PTSD	posttraumatic stress disorder
EAS	equine-assisted services
EAL	equine-assisted learning
PIH	psychotherapy incorporating horses
PCL-5	PTSD Checklist for DSM-5
AAQII	Acceptance and Action Questionnaire II
PANAS	Positive and Negative Affect Scale
STAI	State-Trait Anxiety Inventory
BDI	Beck Depression Inventory
PTGI	Posttraumatic Growth Inventory
FDR	false discovery rate
PF	psychological flexibility
